# 7,8-Dihydroxyflavone Protects Nigrostriatal Dopaminergic Neurons from Rotenone-Induced Neurotoxicity in Rodents

**DOI:** 10.1155/2019/9193534

**Published:** 2019-03-03

**Authors:** Shuke Nie, Kai Ma, Mingkuan Sun, Matthew Lee, Yang Tan, Guiqin Chen, Zhentao Zhang, Zhaohui Zhang, Xuebing Cao

**Affiliations:** ^1^Department of Neurology, Renmin Hospital of Wuhan University, Wuhan 430060, China; ^2^Department of Neurology, Union Hospital, Tongji Medical College, Huazhong University of Science and Technology, Wuhan 430022, China; ^3^Department of Pathology, Johns Hopkins University School of Medicine, Baltimore, MD 21205, USA; ^4^Whiting School of Engineering, Johns Hopkins University, Baltimore, MD 21205, USA

## Abstract

7,8-Dihydroxyflavone (7,8-DHF) is thought to be a promising therapeutic agent for various neurodegenerative diseases. The major purpose of this study was to investigate the neuroprotective effects of 7,8-DHF on the rotenone-induced motor deficit of Parkinson's disease. Nine-month-old rats were treated with rotenone (2 mg/kg/day, i.h.) for 5 weeks to establish the animal model of Parkinson's disease (PD), and 7,8-DHF (5 mg/kg, i.p.) was administrated daily throughout the whole period of rotenone injection. Five weeks later, an open field test was used to assess the motor ability of the animals. TH immunostaining was performed to evaluate rotenone-induced neurotoxicity on substantia nigra (SN) dopaminergic neurons and the DA terminals in the striatum. Western blot analyses were used to examine the expressions of TH, BDNF/TrkB signaling cascades, phospho-*α*-synuclein (Ser129), *α*-synuclein, and phospho-tau (Ser396) in SN. The results revealed that treatment with 7,8-DHF improved PD model's behavioral performance and reduced dopaminergic neuron loss in the SN and striatum, associated with the activation of TrkB receptors and its signaling cascades, and reduced *p*-MAPK, *p*-*α*-synuclein, and *p*-tau. Collectively, these results indicated that 7,8-DHF displayed prominent neuroprotective properties, providing a promising therapeutic strategy for PD treatment.

## 1. Introduction

Neurons and glial cells have been shown to synthesize and secrete a wide variety of neurotrophins, such as nerve growth factor (NGF) and brain-derived neurotrophic factor (BDNF), and play an important role in the survival of neurons and the growth of neurites [1]. The expression of BDNF is upregulated after a traumatic brain injury [2]. Tremendous basic and clinical studies have confirmed the essential role of BDNF in the survival of dopamine neurons and the pathophysiological mechanisms of PD [3, 4]. The level of serum BDNF in patients with PD was significantly lower than those in normal subjects, and the decreased level of serum BDNF was closely related to the degree of apoptosis in striatal dopaminergic neurons [5]. Meanwhile, decreased BDNF levels in the substantia nigra, dentate nucleus, and putamen of patients with PD further proved that BDNF plays an important role in the pathogenesis and development of PD [6]. BDNF activates the specific binding receptor, tyrosine receptor kinase B (TrkB), on neuron membranes to exert its biological effects, which are then distributed to many parts of the brain such as the cortex, hippocampus, dentate gyrus, striatum, septum, and spinal cord [7]. After specific binding to BDNF, the TrkB receptor undergoes autophosphorylation, further activating signaling pathways downstream such as Ras-MAPK, PI3K/Akt, and PLC-*γ* and exerting protective effects. However, clinical trials with recombinant BDNF were disappointing. Presumably, these results are due to its short half-life, which prevents its passage through the blood-brain barrier (BBB) [8]. To circumvent the pharmacokinetic curbs of BDNF, several small molecule chemicals such as deoxygedunin, LM22A, and N-acetylserotonin that are able to pass through the BBB have been demonstrated to mimic the role of BDNF and activate the specific receptor TrkB, exerting neuroprotective effects [9–11].

Our previous study showed that deoxygedunin as a potent TrkB agonist displays remarkable neuroprotective effects in the 6-OHDA-lesioned rat model and MPTP-lesioned mouse model of PD independently of BDNF [12]. Other TrkB agonists including N-acetylserotonin (NAS) derivatives such as HIOC and 7,8-dihydroxyflavone (7,8-DHF) have been widely investigated in the neurodegenerative disease [9, 11, 13–15]. 7,8-DHF and NAS share the same binding pocket on TrkB ECD, and it is different from the motif on TrkB ECD of dexoygedunin [9]. Administration of 7,8-DHF (5 mg/kg) ameliorated nigrostriatal dopaminergic neurons loss and damage to striatal fibers in the MPTP-induced PD model by decreasing the level of apoptosis protein caspase-3 [13]. Compared to deoxygedunin, 7,8-DHF has lower economic costs, better availability, and greater clinical development and transapplication prospects. However, the neuroprotective effects and mechanisms of 7,8-DHF on the rotenone-induced PD animal model are still unknown.

## 2. Materials and Methods

### 2.1. Animals

Thirty-six male Sprague–Dawley rats (220–250 g) were obtained from HFK Bioscience Company (Beijing, China). Animals were kept at 21–23°C with a 12 h light : dark cycle, and food and water available ad libitum. All of the procedures were approved and carried out in accordance with the Rules of Animal Care and Use Committees of Huazhong University of Science and Technology (HUST). Maximum efforts were made to limit the number of animals used and to minimize discomfort. Animals were randomly classified into three groups: control group (*n*=10), rotenone group (*n*=13), and 7,8-DHF group (*n*=13). Animals in the rotenone group and the 7,8-DHF group were injected with rotenone (2 mg/kg, i.h.), daily for 5 weeks. Rats in the 7,8-DHF group were administered 7,8-DHF (5 mg/kg, i.p.) once a day for 5 weeks. The other two groups were given an equal volume vehicle. Three animals in the rotenone group and one animal in the 7,8-DHF group were excluded from the study and were euthanized using CO_2_ followed by decapitation, due to sharp weight loss caused by the toxicity of rotenone.

### 2.2. Materials

Rotenone was purchased from Sigma-Aldrich (USA). 7,8-DHF was obtained from Tokyo Chemical Industry (Japan). The configuration method of rotenone was as follows: rotenone : DMSO : corn oil = 100 mg : 1 ml : 49 ml, prepared a working solution of concentration 2 mg/ml and stored in the dark. One percent sodium pentobarbital was purchased from Wuhan Union Hospital (China). Rabbit polyclonal tyrosine hydroxylase antibody (ab112), rabbit monoclonal BDNF antibody (ab108383), rabbit anti-TrkB (ab33655), and rabbit anti-phospho-TrkB (Y816) (ab75173) antibodies were purchased from Abcam company. Rabbit p44/42 MAPK(Erk1/2) mAb (no. 4695), rabbit phospho-p44/42 MAPK(Erk1/2) mAb (no. 4370), rabbit Akt (pan) mAb (no. 4691), rabbit phospho-Akt (Ser473) mAb (no. 4060), rabbit anti-phospho-tau (S396) antibody, mouse anti-*β*-actin (8H10D10) antibody (no. 3700), and HRP-conjugated secondary anti-rabbit or anti-mouse antibody were purchased from Cell Signaling Technology. Goat anti-rabbit antibody (Alexa Fluor 488) was purchased from Thermo Fisher company. Rabbit anti-*p*-*α*-synuclein (Ser129) antibody and mouse anti-*α*-synuclein antibody were obtained from Genetex company. DAB kit was purchased from Dako Omnis (K5007). Other chemicals and reagents not included above were purchased from Sigma-Aldrich.

### 2.3. Behavioral Test

An open field test was used to assess the motor ability of the animals 5 weeks after modeling of rotenone and intervention of 7,8-DHF. One hour after the last administration of 7,8-DHF, animals in all the three groups were subject to behavioral experiments. This method aims at assessing the behavioral activity and autonomous activity of the PD model. The total distance and trajectory of each rat's movement in 5 minutes was determined. Longer distance and higher average speed within the 5 minutes indicated greater activity in the animal being tested.

### 2.4. Tissue Preparation

After the open field behavioral test, for IHC and IF staining, animals in the three groups were lethally anesthetized with 1% sodium pentobarbital and were transcardially perfused with saline plus 4% paraformaldehyde solution for 8 min. Brains were dissected and immersed overnight in 4% PFA at 4°C. Striatum and SN were cut into 3 mm thick coronal slices using a rodent brain matrix (RWD Life Science, China). Brain slice contained striatum, and SN was processed for paraffin embedding and then was routinely sliced into 5 *μ*m sections. For the western blot analysis, animals were euthanized in a CO_2_ chamber followed by decapitation. Subsequently, SN and striatum were dissected out immediately after death, frozen in liquid nitrogen, and stored at −80°C.

### 2.5. Immunofluorescent and Immunohistochemistry Stainings

The presence of TH-positive neurons in SN of animals was evaluated by IF. Distributions of dopaminergic neuron terminal densities in the striatum and phosphorylated-TrkB neurons in SN were detected by IHC. Briefly, paraffin-embedded slides were deparaffinized through xylene and rehydrated in 95% and 100% alcohol. Rinse slides in running water for 5 min. Antigen retrieval was performed using a microwave for 20 mins in 0.01 M citrate buffer (PH 6.0). Endogenous peroxidases were quenched by immersing slides in 0.3% H_2_O_2_ for 30 mins and then washed three times in distilled water. All sections were blocked overnight in 3% BSA, 1XPBS, and 0.3% Triton X-100 at room temperature for 30 min. After blocking, slides were incubated with specific primary antibodies (rabbit polyclonal tyrosine hydroxylase primary antibody (1 : 500, Abcam) and anti-phosphorylated-TrkB (Y816) (1 : 1000, Abcam)) overnight at 4°C. Wash slides three times with 1X PBST (5 min each). For IHC, slides were incubated with HRP-conjugated secondary antibody at room temperature for 1 hour and then visualized using DAB solution (Vector Laboratories, CA). After haematoxylin-counterstain, dehydration, and mounting, bright-field images were captured by the Olympus camera connected to the microscope. Image J software was used to analyze the images. For example, for the optical density analysis of *p*-TrkB-positive neurons in SN and TH-positive terminal in the striatum, we used a “Colour Deconvolution” plugin developed by Gabriel Landini in Image J, choosing the “Haematoxylin and DAB” vector to separate haematoxylin from DAB, and then obtained the DAB image for analysis of optical density. For IF, after washing with 1X PBST, tissues were incubated with secondary antibody (fluorescence) for 2 hours at room temperature in a humidified chamber. Finally, slides were mounted using Vector mounting medium containing DAPI. TH-positive neurons of IF were counted as previously described [12].

### 2.6. Brain Protein Extraction and Western Blot Analysis

SN tissues in each group were homogenized in RIPA buffer containing protease inhibitor PMSF (PMSF : RIPA = 1 : 99), cocktail (Roche, Canada), and phosphatase inhibitor PhosStop (Roche, Canada) on ice. After centrifugation at 12,000 g for 15 min at 4°C, the supernatants were saved for analysis. Protein concentrations of RIPA fractions were determined using the BCA (P0012, Beyotime company, China), and sample concentration was adjusted equally with the lysis buffer. Collected supernatants were boiled in 5X SDS loading buffer with the proportion of 4 : 1. After SDS-PAGE and transmembrane, the blots were blocked with 5% fat-free milk in TBST for 2 hours at room temperature, and then incubated with primary antibody overnight at 4°C: rabbit tyrosine hydroxylase antibody (1 : 800, Genetex), rabbit BDNF antibody (1 : 1000, Abcam), rabbit polyclonal TrkB antibody (1 : 2000, Abcam) and rabbit phospho-TrkB (Y816) antibody (1 : 1000, Abcam), rabbit monoclonal p44/42 MAPK(Erk1/2) antibody (1 : 2000, Cell Signaling), rabbit phospho-p44/42 MAPK(Erk1/2) antibody (1 : 2000, Cell Signaling), rabbit phospho-tau (S396) antibody (1 : 1000, Cell Signaling), rabbit Akt antibody (1 : 1000, Cell Signaling), rabbit phospho-Akt (Ser473) antibody (1 : 2000, Cell Signaling), rabbit *p*-*α*-synuclein (Ser129) antibody (1 : 1000, Genetex), mouse *α*-synuclein antibody (1 : 500, Genetex), and mouse *β*-actin antibody (1 : 1000, Cell Signaling). After being washed three times with TBST, the blots were incubated with secondary anti-rabbit (1 : 5000, Cell Signaling) or anti-mouse antibodies (1 : 4000, Cell Signaling) for 2 hours at room temperature. After washing with TBST, the blots were incubated with ECL for 2 minutes and then exposed using the Bio-Rad imaging system. The western blot bands were analyzed with Image J software. Relative band intensities were calculated as a ratio of the phosphorylated protein to total protein for TrkB, Erk1/2, Akt, and *α*-synuclein. Relative band intensities of TH, BDNF, and *p*-tau (S396) were measured with *β*-actin, serving as an internal control.

### 2.7. Statistical Analysis

The data were presented as mean ± SEM. Statistical analysis was carried out by SPSS 20 software. Student's *t*-tests were used when only two groups were compared. Statistical evaluation was analyzed with a one-way analysis of variance (ANOVA) followed by Tukey's multiple comparison tests (more than two groups). The significance level was set to *P* < 0.05.

## 3. Results

### 3.1. 7,8-DHF Improves Behavior Performance in Rotenone-Induced PD Animal Model

**Figure 1:**
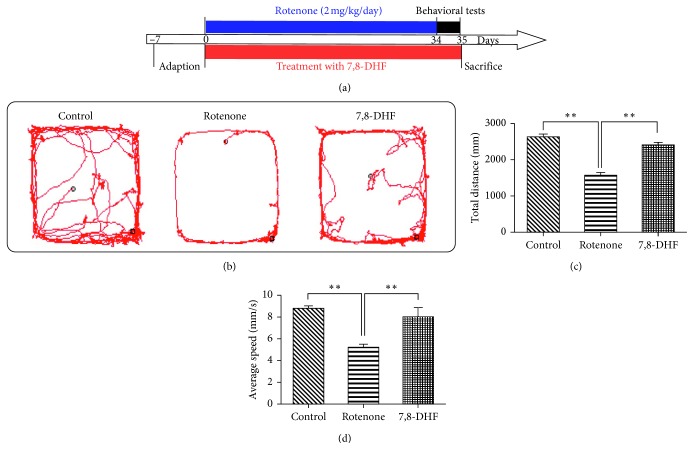
Effect of 7,8-DHF on the open field test behavior of the rotenone-induced rat model. (a) Schematic representation of the experiment timeline. (b–d) 7,8-DHF improves autonomous activity in rotenone-treated rats. Compared to the control group (*n*=10), the total distance of autonomous exercise and the average speed of the rats in the rotenone group (*n*=10) were significantly reduced (^*∗∗*^*p* < 0.01). Treatment with 7,8-DHF (*n*=12) for five weeks prevented this decrease, suggesting a reversion of motor deficit (^*∗∗*^*p* < 0.01). Data were presented as mean ± SEM.

It has been well documented that BDNF is a useful therapeutic agent for PD [16]. To further determine whether 7,8-DHF exhibits any therapeutic effects on PD, we employed a rotenone-induced PD rat model. Five weeks after modeling, an open field test was used to evaluate the autonomous activities of rats in the three groups. The total distance and average speed of movement of each rat within 5 min in all groups were determined. Compared to the control groups, the total distance of autonomous exercise and the average speed of rats in the rotenone group were significantly reduced, indicating impaired movement abilities (distance: 2637.59 ± 69.38 vs 1573.73 ± 77.91 mm, *p* < 0.01; average speed: 8.79 ± 0.23 vs 5.25 ± 0.26 mm/s, *p* < 0.01) (Figure 1). There were statistically significant differences in the distance of autonomous exercise and the average speed between the rotenone group and the 7,8-DHF group (distance: 1573.73 ± 77.91 vs 2413.73 ± 63.10 mm, *p* < 0.01; average speed: 5.25 ± 0.26 vs 8.04 ± 0.21 mm/s, *p* < 0.01), suggesting the protection of locomotion function with 7,8-DHF treatment.

### 3.2. 7,8-DHF Protects Dopaminergic Neurons in a Rotenone-Induced PD Rat Model

**Figure 2:**
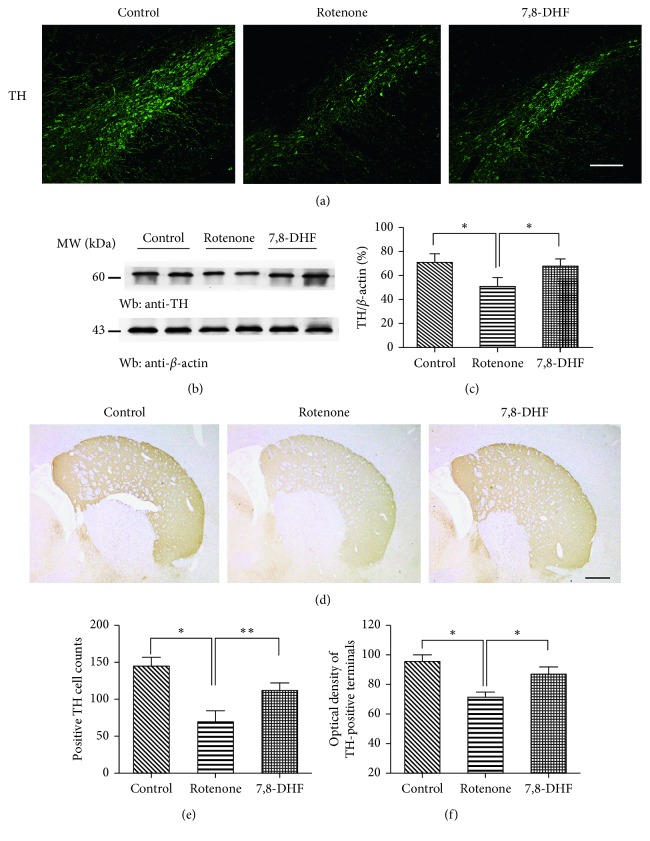
Tyrosine hydroxylase immunostaining of substantia nigra (SN) and striatum in the rotenone-induced PD model. (a) Immunofluorescence of tyrosine hydroxylase in the substantia nigra pars compacta (SNpc). Scale bar = 100 *μ*m (up). We found that there were fewer TH-positive neurons in the similar anatomic level sections of SN in the rotenone group than the control group. Compared to the rotenone group, treatment with 7,8-DHF significantly attenuated the loss of dopaminergic neurons in SNpc (a, e). The western blots of TH in SN further reconfirmed the findings (b, c). ^*∗*^*p* < 0.05, ^*∗∗*^*p* < 0.01, a significant difference between the two indicated groups. The administration of rotenone also resulted in a decrease of TH-positive DA terminals in the striatal region of animals in the rotenone group compared to the 7,8-DHF group (d, f). Scale bar = 100 *μ*m (down). Data were presented as mean ± SEM *n*=4.

After the behavioral test, the integrity of dopaminergic neurons in the SNpc and terminal fibers in the striatum was evaluated by immunofluorescent and immunohistochemistry with a dopaminergic neuron-specific marker, anti-TH antibody. We found that there were fewer TH-positive neurons in the similar anatomic level sections of SN in the rotenone group than in normal rats (70.17 ± 14.93 vs 145.67 ± 12.01, *p* < 0.05) (Figures 2(a) and 2(e)). This observation was quantified by cell counting in a double-blind way and confirmed by western blots of the SN lysates (Figures 2(b) and 2(c)). Cell counts also indicated statistically significant preservation of TH-positive dopaminergic SN neurons in the 7,8-DHF group than the rotenone group (112.58 ± 10.27 vs 70.17 ± 14.93, *p* < 0.01) (Figures 2(a) and 2(e)). Western blots of TH in SN further confirmed these findings. The ratio of TH/*β*-action in the 7,8-DHF group was significantly higher than the rotenone group (67.65 ± 6.56% vs 51.14 ± 7.18%, *p* < 0.05). Compared to the control group, the striatum resulted in a decrease of the TH-positive DA terminals in the striatal region (section on bregma +2.5) of the rotenone group (94.58 ± 4.52 vs 70.78 ± 3.19, *p* < 0.05) (Figures 2(d) and 2(f)), and 7,8-DHF could reverse the reduction of dopaminergic terminals in the striatum. Hence, the treatment with 7,8-DHF resulted in noticeable protective effects on the striatal TH staining.

### 3.3. 7,8-DHF Activates TrkB Signaling and Reduces the Abnormal Phosphorylation of *α*-Synuclein and Tau

**Figure 3:**
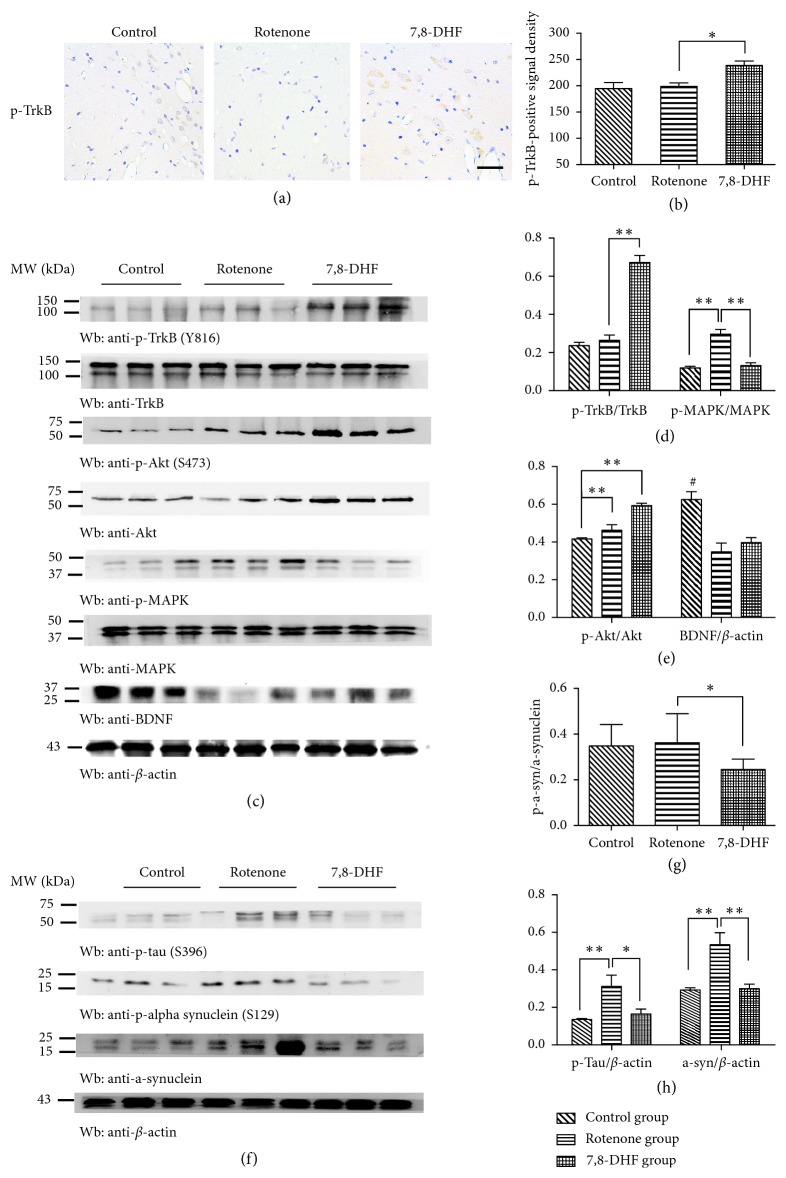
7,8-DHF activates TrkB signaling and reduces the abnormal phosphorylation of MAPK, *α*-synuclein, and tau. TrkB phosphorylation in SNpc regions was significantly elevated in the 7,8-DHF group compared to the rotenone (*p* < 0.05), suggesting 7,8-DHF activated TrkB markedly (a, b). Scale bar = 50 *μ*m, ^*∗*^*p* < 0.05, compared to the two indicated groups, *n*=4. *p*-TrkB and *p*-Akt in the 7,8-DHF group were significantly higher than other two groups, and there was no significant difference in the expression of BDNF in the SN between rotenone and 7,8-DHF groups (c, e). ^*∗∗*^*p* < 0.01, compared to other two groups, *n*=4. The phosphorylated MAPK (*p*-MAPK) in the rotenone group was significantly increased compared to the control group, and treatment with 7,8-DHF reduced *p*-MAPK (c, d). The expressions of *α*-synuclein and *p*-tau (S396) in the rotenone group were significantly higher compared to the control group, and 7,8-DHF treatment could reduce the levels of total *α*-synuclein, phospho-*α*-synuclein (Ser 129), and *p*-tau (S396) (f–h), (^*∗*^*p* < 0.05, ^*∗∗*^*p* < 0.01, compared to the two indicated groups, *n*=4). Data were presented as mean ± SEM.

The activation of TrkB through its autophosphorylation likely contributed to its neuroprotective roles. Thus, it is vital to demonstrate whether treatment with 7,8-DHF could activate TrkB. We performed immunohistochemistry to examine TrkB activation in SN. Rat brain sections containing the SNpc regions were stained with *p*-TrkB (Y816) antibody to evaluate the activation of the TrkB receptor. As shown in Figures 3(a) and 3(b), TrkB phosphorylation in SNpc regions was significantly elevated in the 7,8-DHF group compared to the rotenone group (238.6 ± 4.251 vs 199.0 ± 3.305, *p*=0.0286), indicating that 7,8-DHF treatment activates TrkB markedly. As expected in the western blot analysis, the TrkB receptors were more prominently phosphorylated in the 7,8-DHF group than the rotenone group (67.24 ± 3.67% vs 26.41 ± 2.79%, *p* < 0.01), as well the downstream pathways involving PI3K/Akt pathway (59.29 ± 1.26% vs 46.21 ± 2.97%, *p* < 0.05), and there was no significant difference in the expression of BDNF in the SN between rotenone group and 7,8-DHF group (34.78 ± 4.63% vs 39.67 ± 2.59%, *p* > 0.05) (Figures 3(c) and 3(e)). The quantitative ratios of *p*-TrkB/TrkB, *p*-Akt/Akt, and BDNF/*β*-actin are summarized in Figure 3. Noticeably, this effect by 7,8-DHF was independent of altering BDNF expression in the animals (Figure 3(e)). Combining with the behavioral tests and immunohistochemistry staining results, we concluded that treatment with 7,8-DHF activates TrkB receptors and downstream signaling pathways in a BDNF-independent manner. Meanwhile, the phosphorylation of MAPK in the rotenone group was significantly increased compared to the control group (29.62 ± 2.46% vs 11.89 ± 0.84%, *p* < 0.01), and treatment with 7,8-DHF could downregulate the expression level of *p*-MAPK/MAPK (13.20 ± 1.47% vs 29.62 ± 2.46%, *p* < 0.01) (Figures 3(c) and 3(d)), suggesting 7,8-DHF affected the phosphorylation of MAPK independent of the BDNF/TrkB pathway and conferred neuroprotection against rotenone-induced neurological dysfunction via its other biological functions which would be discussed later.

Tauopathy and *α*-synucleinopathy are hallmark features that had been demonstrated in tremendous studies of Parkinson's disease [17]. As expected in the subsequent analysis of western blotting, phosphorylated tau (S396) in the rotenone group was significantly increased compared to the control group (*p*-tau: 31.20 ± 5.95% vs 13.43 ± 0.67%, *p* < 0.01); however, no significant difference was found with the ratio of phosphorylated *α*-synuclein (S129)/*α*-synuclein in the rotenone group compared to the control group (*p*-*α*-synuclein/*α*-synuclein: 36.21 ± 4.24% vs 34.83 ± 3.12%, *p* > 0.05). It is intriguing that treatment with 7,8-DHF can reduce pathological hyperphosphorylated tau and *α*-synuclein compared to the rotenone group (*p*-*α*-synuclein/*α*-synuclein: 24.52 ± 4.56% vs 36.21 ± 4.24%, *p* < 0.05; *p*-tau: 16.43 ± 2.62% vs 31.20 ± 5.95%, *p* < 0.01) (Figures 3(f) and 3(g)), suggesting that 7,8-DHF could ameliorate the *α*-synucleinopathy and tauopathy in the rotenone-induced animal model of PD.

### 3.4. Effect of 7,8-DHF on the Peripheral Organs of the Rotenone-Induced Rat Model

**Figure 4:**
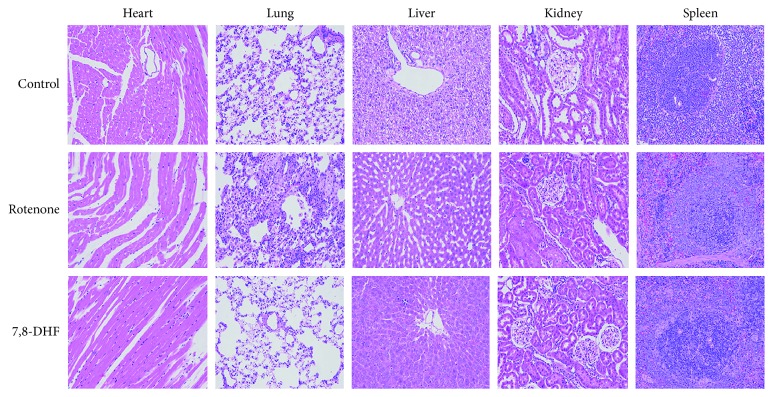
Effect of 7,8-DHF on the peripheral organs of the rotenone-induced PD animal model. Pathological changes in peripheral organs of rats exposed to rotenone, based on H&E staining, presented alveolar ectasia and alveolar wall fragmentation in the lung and hepatic sinus expansion around the central tube in the liver. No associated peripheral organs toxicity was observed in the heart, kidney, and spleen.

The toxicity of rotenone on the peripheral organs varies depending on the dosage and time and route of administration. Previous research showed the subcutaneous infusion of rotenone (3 mg/kg/day, 3–5 days) failed to produce dopaminergic lesions but led to extensive peripheral organ toxicity. Meanwhile, intracerebral infusion (5.0 *μ*g/day, 28 days) of this toxin produced a progressive lesion of the nigrostriatal dopaminergic pathway over 28 days with no associated peripheral toxicity [18]. In order to evaluate the toxicity of rotenone in our animal models, HE staining was performed to explore the pathology of the peripheral organs (heart, lung, liver, kidney, and spleen). Compared to the control and 7,8-DHF groups, significant pathologic changes were observed in the lung and liver of rats in the rotenone group. While no obvious changes were found in the kidney, heart, and spleen, rats in the rotenone group presented alveolar ectasia and alveolar wall fragmentation in the lung and hepatic sinus expansion around the central tube in the liver, suggesting that 7,8-DHF could protect peripheral organs against rotenone toxicity (Figure 4) with its anti-inflammation and antioxidant properties [19, 20]. Mortality of animals in rotenone and 7,8-DHF groups was associated with rotenone toxicity in the five weeks' modeling period.

## 4. Discussion

This study showed that 7,8-DHF could attenuate motor deficit of the rotenone-induced PD model and exert neuroprotective effects through activating the TrkB receptor and the downstream signaling Akt and reducing the abnormal phosphorylation of MAPK, *α*-synuclein, and tau. Our previous study had demonstrated another small molecule deoxygedunin protected nigrostriatal dopaminergic neurons from MPTP and 6-OHDA-induced neurotoxicity [12]. Compared to MPTP and 6-OHDA, rotenone as a pesticide used in this study has been demonstrated to induce misfolding and aggregation of *α*-synuclein, partially replicating *α*-synucleinopathy in patients with PD [21]. Meanwhile, the intervention drug-7,8-DHF in the present study has better translational medicine prospect due to its fully verified neuroprotection effect [22–24].

In vivo pharmacokinetic profiles and metabolism of 7,8-DHF and its metabolites distribute in mouse and monkey brains after oral administration had been well researched [25–27]. It had been identified that the concentration of 7,8-DHF both peaked at 10 min with 70 ng/ml in the plasma and 50 ng/g in the brain after the administration of 7,8-DHF (5 mg/Kg). What is more, 7,8-DHF was still detectable in the plasma even at 8 h with a concentration of 5 ng/ml. Meanwhile, 7,8-DHF and its synthetic derivative 4'-DMA-7,8-DHF provoked TrkB activation at 1-2 hours, and the stimulatory effect is demonstrable even at 4 h in the mouse brain [25]. The half-life of 7,8-DHF in the monkey plasma is about 4–8 hours, and monkeys maintain healthy state throughout the whole course of seven-month treatment with 7,8-DHF (30 mg/kg/day) [26]. Long half-time period and favorable oral bioavailability support the fact that 7,8-DHF is a superior compound for the drug development of neurodegenerative disease. In the present study, a behavioral test was performed one hour after the last administration of 7,8-DHF, and the tissues were harvested within the following four hours, indicating animals were all under the influence of this drug when samples were collected.

7,8-DHF has been recognized as a potential pharmacotherapeutic strategy for PD, AD, Huntington's disease, diet-induced obesity, fragile X syndrome, and traumatic brain injury [15, 22–24, 28]. Previous studies have focused on its high affinity and specificity with BDNF's specific receptor TrkB and the activation of BDNF/TrkB downstream signaling cascade. It is noted that this compound could also regulate the synapse expression of AMPA receptors in an animal model of fragile X syndrome and reduce reactive oxygen species (ROS) production caused by glutamate in vitro [20, 22]. Several pieces of research published recently found that 7,8-DHF and various other reported small-molecule TrkB agonists might not actually be direct agonists of the TrkB and might be mediating their observed effects by other mechanisms [29, 30]. In this study, we also found that 7,8-DHF affected the phosphorylation of MAPK independent of the BDNF/TrkB pathway, indicating that 7,8-DHF conferred neuroprotection against rotenone-induced neurological dysfunction via its other biological functions. Elevated phosphorylation of MAPK had been demonstrated in the patients with AD and Down syndrome, suggesting that it may be involved in the pathogenesis of the disease [31, 32]. In the 6-hydroxydopamine- (6-OHDA-) lesioned rat model, the level of phosphorylated *p*-MAPK was revealed to be associated with the levels of BDNF and GDNF in striatum and substantia nigra among different postlesion time points, and one major point was that there was no significant change in the expression of phosphorylated Akt at the same time points [33]. In the present study, the expression of BDNF in the substantia nigra of animals in the rotenone group was less than the control group, leading to the downregulation of the expression of phosphorylated MAPK. Rotenone as a neurotoxic agent had been found to promote the hyperphosphorylation of *p*-ERK1/2 in the animal model of the neurodegenerative disease [34]. In the above study, the expression level of *p*-ERK1/2 was enhanced evidently in the cultured neurons treated by rotenone, subsequently decreased significantly with pretreatment of PD98059 and sulindac sulfide (a nonsteroidal anti-inflammatory drug) [34]. However, the concrete mechanism of rotenone on the phosphorylation of ERK is still unclear.

ERK could be activated and phosphorylated by reactive oxygen species (ROS) and inhibit the phosphorylation of ERK1/2, reducing ROS-induced cell death [35]. Intriguingly, rotenone, as a mitochondrial complex-I inhibitor, was able to induce apoptosis by enhancing the amount of mitochondrial ROS production [36]. So, ROS and subsequently related involvement of oxidative damage should be the important mechanisms of toxicity in rotenone models of PD [37]. In the present study, the level of phosphorylated MAPK in the substantia nigra of animals in the rotenone group increased after 35 days of subcutaneous injection, which was consistent with previous studies. What is more, 7,8-DHF had been demonstrated to reduce ROS production, providing neuroprotection against glutamate-induced toxicity [20, 38]. Taken together, we believe 7,8-DHF could decrease the phosphorylation of *p*-MAPK by reducing the ROS production via its antioxidant activity, suggesting the possible mechanism of increased expression of phosphorylated MAPK in the rotenone group and the reduced level of this protein in the 7,8-DHF group.

Abnormal aggregation of hyperphosphorylated tau was observed in MPTP, paraquat, and rotenone-induced PD models, and the expression level of *α*-synuclein was significantly increased [39, 40]. In the 6-OHDA-induced PD rat model, it was found that decreased tau levels did not improve the neurotoxic damage of 6-OHDA in the animal model of PD, which may not alert abnormal aggregation of *α*-synuclein and production of LBs, lacking interaction with homogenous *α*-synuclein [41]. Phosphorylated tau had been found only to increase the folding modification of *α*-synuclein. *α*-synuclein is a necessary condition for provoking phosphorylation and aggregation of tau protein in vitro, showing that *α*-synuclein may trigger the initiation of tauopathy [42]. In this study, we found that rotenone upregulated the levels of both *α*-synuclein and phosphorylated tau (S396), indicating there might be an interaction between these two proteins in the pathogenesis of PD. Treatment with 7,8-DHF could reduce the hyperphosphorylation of the two proteins. However, the specific mechanism still needs to be further explored. This study had some limitations that have to be pointed out. Although significant motor impairment had been demonstrated in the open field test in various PD models [43], only one behavioral test is not sufficient to reflect the motor function of the animal model of PD, and other behavioral tests such as rotation test and beam traversal test should be evaluated in the future studies. Meanwhile, the activity of ROS should be investigated in the later study in order to better explain the mechanism underlying the increased *p*-MAPK in the rotenone group and reduced *p*-MAPK following by the treatment with 7,8-DHF.

Collectively, neuroprotective effects of 7,8-DHF on the classical subacute animal models of PD have been investigated in this study. Our study provides not only as a useful tool to explore the molecular mechanism of BDNF/TrkB signaling in PD but also as a promising therapeutic agent for the treatment of this disease.

## Data Availability

The data used to support the findings of this study have been deposited in the figshare repository (https://doi.org/10.6084/m9.figshare.6959159).

## References

[1] Huang E. J., Reichardt L. F. (2001). Neurotrophins: roles in neuronal development and function. *Annual Review of Neuroscience*.

[2] Rostami E., Krueger F., Plantman S. (2014). Alteration in BDNF and its receptors, full-length and truncated TrkB and p75NTR following penetrating traumatic brain injury. *Brain Research*.

[3] Nagahara A. H., Tuszynski M. H. (2011). Potential therapeutic uses of BDNF in neurological and psychiatric disorders. *Nature Reviews Drug Discovery*.

[4] Xu H., Belkacemi L., Jog M., Parrent A., Hebb M. O. (2013). Neurotrophic factor expression in expandable cell populations from brain samples in living patients with Parkinson’s disease. *FASEB Journal*.

[5] Scalzo P., Kümmer A., Bretas T. L., Cardoso F., Teixeira A. L. (2009). Serum levels of brain-derived neurotrophic factor correlate with motor impairment in Parkinson’s disease. *Journal of Neurology*.

[6] Parain K., Murer M. G., Yan Q. (1999). Reduced expression of brain-derived neurotrophic factor protein in Parkinsonʼs disease substantia nigra. *NeuroReport*.

[7] Skaper S. (2008). The biology of neurotrophins, signalling pathways, and functional peptide mimetics of neurotrophins and their receptors. *CNS & Neurological Disorders-Drug Targets*.

[8] Thoenen H., Sendtner M. (2002). Neurotrophins: from enthusiastic expectations through sobering experiences to rational therapeutic approaches. *Nature Neuroscience*.

[9] Jang S.-W., Liu X., Chan C. B. (2010). Deoxygedunin, a natural product with potent neurotrophic activity in mice. *PLoS One*.

[10] Massa S. M., Yang T., Xie Y. (2010). Small molecule BDNF mimetics activate TrkB signaling and prevent neuronal degeneration in rodents. *Journal of Clinical Investigation*.

[11] Liu X., Obianyo O., Chan C. B. (2014). Biochemical and biophysical investigation of the brain-derived neurotrophic factor mimetic 7,8-dihydroxyflavone in the binding and activation of the TrkB receptor. *Journal of Biological Chemistry*.

[12] Nie S., Xu Y., Chen G. (2015). Small molecule TrkB agonist deoxygedunin protects nigrostriatal dopaminergic neurons from 6-OHDA and MPTP induced neurotoxicity in rodents. *Neuropharmacology*.

[13] Jang S.-W., Liu X., Yepes M. (2010). A selective TrkB agonist with potent neurotrophic activities by 7,8-dihydroxyflavone. *Proceedings of the National Academy of Sciences*.

[14] Wang B., Wu N., Liang F. (2013). 7,8-dihydroxyflavone, a small-molecule tropomyosin-related kinase B (TrkB) agonist, attenuates cerebral ischemia and reperfusion injury in rats. *Journal of Molecular Histology*.

[15] Zhang Z., Liu X., Schroeder J. P. (2013). 7,8-dihydroxyflavone prevents synaptic loss and memory deficits in a mouse model of Alzheimer’s disease. *Neuropsychopharmacology*.

[16] Baquet Z. C., Bickford P. C., Jones K. R. (2005). Brain-derived neurotrophic factor is required for the establishment of the proper number of dopaminergic neurons in the substantia nigra pars compacta. *Journal of Neuroscience*.

[17] Spires-Jones T. L., Attems J., Thal D. R. (2017). Interactions of pathological proteins in neurodegenerative diseases. *Acta Neuropathologica*.

[18] Ravenstijn P. G. M., Merlini M., Hameetman M. (2008). The exploration of rotenone as a toxin for inducing Parkinson’s disease in rats, for application in BBB transport and PK-PD experiments. *Journal of Pharmacological and Toxicological Methods*.

[19] Park H. Y., Kim G. Y., Hyun J. W. (2012). 7,8-Dihydroxyflavone exhibits anti-inflammatory properties by downregulating the NF-κB and MAPK signaling pathways in lipopolysaccharide-treated RAW264.7 cells. *International Journal of Molecular Medicine*.

[20] Chen J., Chua K.-W., Chua C. C. (2011). Antioxidant activity of 7,8-dihydroxyflavone provides neuroprotection against glutamate-induced toxicity. *Neuroscience Letters*.

[21] Silva B., Einarsdóttir Ó., Fink A., Uversky V. (2013). Biophysical characterization of α-synuclein and rotenone interaction. *Biomolecules*.

[22] Tian M., Zeng Y., Hu Y. (2015). 7,8-Dihydroxyflavone induces synapse expression of AMPA GluA1 and ameliorates cognitive and spine abnormalities in a mouse model of fragile X syndrome. *Neuropharmacology*.

[23] Zhao S., Gao X., Dong W., Chen J. (2015). The role of 7,8-dihydroxyflavone in preventing dendrite degeneration in cortex after moderate traumatic brain injury. *Molecular Neurobiology*.

[24] Garcia-Diaz Barriga G., Giralt A., Anglada-Huguet M. (2017). 7,8-dihydroxyflavone ameliorates cognitive and motor deficits in a Huntington’s disease mouse model through specific activation of the PLCγ1 pathway. *Human Molecular Genetics*.

[25] Liu X., Qi Q., Xiao G., Li J., Luo H. R., Ye K. (2013). O-methylated metabolite of 7,8-dihydroxyflavone activates TrkB receptor and displays antidepressant activity. *Pharmacology*.

[26] He J., Xiang Z., Zhu X. (2016). Neuroprotective effects of 7,8-dihydroxyflavone on midbrain dopaminergic neurons in MPP+-treated monkeys. *Scientific Reports*.

[27] Sun T., Chen S., Huang H., Li T., Yang W., Liu L. (2017). Metabolic profile study of 7,8-dihydroxyflavone in monkey plasma using high performance liquid chromatography-tandem mass spectrometry. *Journal of Chromatography B*.

[28] Chan C. B., Tse M. C. L., Liu X. (2015). Activation of muscular TrkB by its small molecular agonist 7,8-dihydroxyflavone sex-dependently regulates energy metabolism in diet-induced obese mice. *Chemistry & Biology*.

[29] Boltaev U., Meyer Y., Tolibzoda F. (2017). Multiplex quantitative assays indicate a need for reevaluating reported small-molecule TrkB agonists. *Science Signaling*.

[30] Todd D., Gowers I., Dowler S. J. (2014). A monoclonal antibody TrkB receptor agonist as a potential therapeutic for Huntington’s disease. *PLoS One*.

[31] Adachi T., Kar S., Wang M., Carr B. I. (2002). Transient and sustained ERK phosphorylation and nuclear translocation in growth control. *Journal of Cellular Physiology*.

[32] Swatton J. E., Sellers L. A., Faull R. L. M., Holland A., Iritani S., Bahn S. (2004). Increased MAP kinase activity in Alzheimer’s and down syndrome but not in schizophrenia human brain. *European Journal of Neuroscience*.

[33] Lui N. P., Chen L. W., Yung W. H., Chan Y. S., Yung K. K. (2012). Endogenous repair by the activation of cell survival signaling cascades during the early stages of rat Parkinsonism. *PLoS One*.

[34] Sai Y., Chen J., Wu Q., Liu H., Zhao J., Dong Z. (2009). Phosphorylated-ERK 1/2 and neuronal degeneration induced by rotenone in the hippocampus neurons. *Environmental Toxicology and Pharmacology*.

[35] Seo S. R., Chong S. A., Lee S.-I. (2001). Zn2+-induced ERK activation mediated by reactive oxygen species causes cell death in differentiated PC12 cells. *Journal of Neurochemistry*.

[36] Li N., Ragheb K., Lawler G. (2002). Mitochondrial complex I inhibitor rotenone induces apoptosis through enhancing mitochondrial reactive oxygen species production. *Journal of Biological Chemistry*.

[37] Sherer T. B., Betarbet R., Testa C. M. (2003). Mechanism of toxicity in rotenone models of Parkinson’s disease. *Journal of Neuroscience*.

[38] Choi J.-Y., Kang J.-T., Park S.-J. (2013). Effect of 7,8-dihydroxyflavone as an antioxidant on in vitro maturation of oocytes and development of parthenogenetic embryos in pigs. *Journal of Reproduction and Development*.

[39] Duka T., Rusnak M., Drolet R. E. (2006). Alpha-synuclein induces hyperphosphorylation of Au in the Mptp model of Parkinsonism. *FASEB Journal*.

[40] Hoglinger G. U., Lannuzel A., Khondiker M. E. (2005). The mitochondrial complex I inhibitor rotenone triggers a cerebral tauopathy. *Journal of Neurochemistry*.

[41] Moussaud S., Jones D. R., Moussaud-Lamodière E. L., Delenclos M., Ross O. A., McLean P. J. (2014). Alpha-synuclein and tau: teammates in neurodegeneration?. *Molecular Neurodegeneration*.

[42] Giasson B. I., Forman M. S., Higuchi M. (2003). Initiation and synergistic fibrillization of tau and alpha-synuclein. *Science*.

[43] Meredith G. E., Kang U. J. (2006). Behavioral models of Parkinson’s disease in rodents: a new look at an old problem. *Movement Disorders*.

